# Supplementary material for: Amino acid substitutions in human growth hormone affect secondary structure and receptor binding
Source: PLoS One. 2023 Mar 23;18(3):e0282741. doi: 10.1371/journal.pone.0282741 (PMC10035860; doi:10.1371/journal.pone.0282741)
Supplement: Attachment — Submitted filename: response-referee-3.docx [file pone.0282741.s005.docx]

### Amino acid substitutions in human growth hormone affect structure and receptor binding

# Response to Referee #3

### – General –

| Reviewer  Comment | Rajkovic et al presents a study of the interaction between the human growth hormone and its receptor. Using a computational mutagenesis method, they generate mutations in the hormone that are designed to improved affinity to the receptor and characterized their effect experimentally. A significant focus is on a particular mutation, E174A, that has been previously found to improve the affinity.  In my view PLOS computational biology is not the optimal choice for this study. The results are mainly experimental. The computational part is limited to running a computational ddG prediction method and this method has been around for 20 years. It is also not possible to describe the computational modeling as successful since none of the mutations increased the affinity beyond the already described E174A.  The introduction describes several broad goals, but these are not backed up by the results in the study. The discussion does not address the goals presented in the introduction. There is also no support for the following statement in the abstract:  “The results point to a link between coiled-coiling, zinc binding, and hGHR-binding affinity in hGH, and also suggest rules for choosing affinity-increasing substitutions.” |
| --- | --- |
| Author  Response | Finding affinity-increasing mutations was the original goal, and indeed it was not reached. For this reason we decided to focus on the mechanism of E174A, but some of the language still reflects the original goal.  We have removed the fragment “and also suggest rules for choosing affinity-increasing substitutions.”  We do think that as attempts to improve affinity one computationally-selected mutation at a time are rare in the literature, our results will help future workers avoid certain pitfalls. |
| Excerpt From  Revised Manuscript |  |

### – E174A –

| Reviewer  Comment | Overall, I have a hard time understanding what the main scientific advancement is in this study. The abstract mainly describes the result for E174A, but this mutation is already described in the literature. There is additional data on kinetics, but those results follows what is expected for affinity-altering mutations (increased off rate). |
| --- | --- |
| Author  Response | E174A was discovered by Cunningham & Wells (Science ‘89), but only KD was measured, so very little was known about the mechanism. Alanine scanning is usually presumed to decrease binding, where it scans through the binding site. The authors found it remarkable enough to include it in the abstract, though all they could say was E174 “hindered” binding. Despite the importance of hGH, 33 years have gone by with no explanation. In this work we connect E174A to monomer structure and zinc binding.  There are two ways to increase affinity: increasing koff, or increasing kon. Prior workers reported only KD, so it was not obvious which one applies. Affinity is not everything, sometimes the goal is to increase koff. So we do think the additional kinetic data are potentially useful. |
| Excerpt From  Revised Manuscript |  |

### – E174A –

| Reviewer  Comment | -       The computational mutagenesis is designed to improve affinity. Yet this was not the happening for any of the mutants except the already described E174A. This needs to be commented on, especially in an article in a computational journal. |
| --- | --- |
| Author  Response | As mentioned above, affinity improvement did not work. We have removed the statement that could be construed as promisory. |
| Excerpt From  Revised Manuscript |  |

### – homodimerization and MD –

| Reviewer  Comment | It is not surprising that it is difficult to predict stabilizing mutations. However, many new methods have been introduced since FoldX was presented 20 years ago. How does other methods perform on your experimental data? |
| --- | --- |
| Author  Response | HomologyScanner was only published last year. Though it is a simple variation on FoldX, it nonetheless provides a significant improvement.  Following the Referee’s suggestion, we now provide the ΔΔG as computed using SSIPe, a recent and well-benchmarked method. This method does not have demonstrably better performance. Part of the issue is that we have deliberately selected based mutants over a narrow range of ΔΔG_homologyScanner,_ also we believe the same issues that challenged FoldX also affect SSIPe. |
| Excerpt From  Revised Manuscript | See revised Table 1.  ..  Discussion  ..  As noted in Table 1, there was no significant correlation between ΔΔG_homologyScanner_ and ΔΔG_experimental_. In addition to the issues mentioned above, the low correlation could also be due to the narrow range in ΔΔG_homologyScanner_ (-2,5 to -0,5 kcal/mol) and small number of mutants (21). Would another method have been better? We tested SSIPe, but this method likewise showed no significant correlation. |

### – Marchand et al. –

| Reviewer  Comment | The protein engineering field has taken large strides over the last decade and it has been demonstrated that protein complexes can be designed from scratch with good affinity already before experimental affinity maturation. The authors use a reference from 2013 to discuss the state-of-art, which does not give a current view of the field. See for example Marchand et al (Curr Opin Struct Biol. 2022 Apr 8;74:102370). |
| --- | --- |
| Author  Response | We thank the Referee for this valuable suggestion and now cite Marchand et al. |
| Excerpt From  Revised Manuscript |  |

### – Coiled-coiling –

| Reviewer  Comment | The growth hormone is not a coiled-coil. It has not, in my view, been demonstrated that the 222/208 ratio can be used for helical bundles to describe individual helix-helix interactions in proteins like is done here. |
| --- | --- |
| Author  Response | The wild type always had a low 222/208 ratio, we clarify that this shows no coiled-coiling in experimental (wild-type) structures.  In E174A the 222/208 ratio increases to a degree which is significant. However we do not claim that all the helices would join coiled-coils, the more likely scenario is a local change. We know of no cause for increases in the 222/208 ratio, other than increases in coiled-coiling. We have reworded our text to better reflect the nuances and cite the relevant literature. We also clarify that further work would be required to determine the nature of the structural change in more detail. |
| Excerpt From  Revised Manuscript |  |

### – Receptor –

| Reviewer  Comment | There is no description of how the receptor is produced. |
| --- | --- |
| Author  Response | It was purchased from AbCam. We now clarify this in our revised Methods. |
| Excerpt From  Revised Manuscript |  |

### – Replicates –

| Reviewer  Comment | There are no error bars on the experimentally determined parameters. Were the measurements replicated? |
| --- | --- |
| Author  Response | We did not intend to suggest our kinetic measurements were highly precise. Rather we grouped comparable variants together. Table 1 shows the variants that with affinity comparable to WT (albeit slower on-rate) in green, to discriminate from a separate group of much-lower affinity mutants. Figure 3 separates out a third group which does not match either of those groups. Though we thought this was clear enough, we will be happy to consider other ways to clarify.  Replicates would be needed if one were to desire greater precision, but as the data were clean and came from an established platform facility, we did not see a need for them. |
| Excerpt From  Revised Manuscript |  |

### – Command line –

| Reviewer  Comment | The command line for FoldX should be included. If a server has been used, this should be indicated. |
| --- | --- |
| Author  Response | Our revised Supplementary Information now includes the FoldX commands. |
| Excerpt From  Revised Manuscript |  |
