# Supplementary material for: Amino acid substitutions in human growth hormone affect secondary structure and receptor binding
Source: PLoS One. 2023 Mar 23;18(3):e0282741. doi: 10.1371/journal.pone.0282741 (PMC10035860; doi:10.1371/journal.pone.0282741)
Supplement: Attachment — Submitted filename: response-referee-2.plosone.docx [file pone.0282741.s006.docx]

### Amino acid substitutions in human growth hormone affect structure and receptor binding

# Response to Referee #2

### – Abstract and Introduction –

| Reviewer  Comment | Reviewer #2: The manuscript by Rajkovic et al. entitled “Amino acid substitutions in human growth hormone affect secondary structure and receptor binding” presents a study of the interaction between the human growth hormone and its receptor. Using a computational and experimental approach to find an action mechanism of 21 discovered mutations of hGH and their impact on the complex with hGHR. The mutations were modeled by FoldX. The mutations expressed and purified were experimentally studied with SPR and CD. About a previously known E174A mutation, the authors propose a mechanistic explanation for the increase in affinity, that involves part of a zinc-binding triad and packed in a coiled-coil interface. The aim of this work is interesting (and this reviewer appreciates it), although there are many inconsistencies between the theoretical and the experimental data that should be addressed in more detail. The paper is not well done in almost all parts, and the manuscript in its present form demands a deep revision before it can be published, so this reviewer suggests to reject the paper. The paper should be modified in some parts. Major issues: 1) The abstract needs to be revised according to the following suggestions. 2) The introduction section is too poor and unclear; nothing is reported on the aim of the paper. |
| --- | --- |
| Author  Response | We appreciate the Referee’s careful reading of our work.  Our original goal was to do in silico affinity maturation; when that did not work as well as we had hoped, we focused on the mechanism of the E174A mutation. E174A rendered a highly counterintuitive result which has not been explained in the 30 years since its discovery at Genentech by Cunningham & Wells. In the revised work we make it much clearer that this is our focus.  While we would not have anticipated the CD result we got, we do not believe it to be inconsistent. We ask the Referee to clarify what part of our paper shows inconsistency between experiment and calculation. |
| Excerpt From  Revised Manuscript | Introduction  Human Growth Hormone (hGH) binds a single hGH Receptor (hGHR) using its Site 1, a large, physicochemically diverse binding region. It then recruits a second hGHR to bind at its lower-affinity Site 2. [1] The hGHR dimerization initiates signaling through the JAK/STAT pathway. Thus one strategy to disrupt signaling is to prevent dimerization. This in turn can be done by destroying binding at site 2, which is easily effected with mutations such as G120K. [2] It is also useful to simultaneously strengthen binding at site 1, [3] and both were done in Pegvisomant development. As part of that process the interesting substitution E174 was discovered which increases binding but whose mechanism could not be explained by Cunningham & Wells. [4] In this work we measure the kinetics and secondary-structural effects of that mutation, along with that of a different substitution at the same position (E174Y), a negative control (L52F, which is positioned far from position 174, and outside the helices), and the Wild Type (WT, the neutral control). |

### – CD –

| Reviewer  Comment | 3) The section Materials and Methods need to be reorganized and reordered. In this way is difficult to read and understand the methodologies used in the paper. About the CD, there are some inconsistencies regards the sample preparation (for example: by 2 cycles of dialysis is not possible to remove all the salt in the sample) |
| --- | --- |
| Author  Response | The sample was 1mL, which we neglected to mention in our earlier submission. We have now corrected this omission in our revised manuscript. The sample was dialyzed against 2L of buffer, in two rounds. We hope the Referee finds the dilution factor of 4 million is sufficient.  We saw no sign that remaining salt was interfering with our measurements. The Gräslund lab was impressed with the clarity of the isodichroic point, this is not often seen outside textbooks. |
| Excerpt From  Revised Manuscript | Circular Dichroism (CD) measurements  CD spectroscopy was used in order to identify the impact of each mutation on rhGH structure. 1 mL of purified rhGH samples was dialyzed against 2 L of 20 mM sodium phosphate buffer at pH 7.2 overnight to remove most of the tris buffer and salt used during purification via 2000-fold dilution. This dialyzed sample was dialyzed a second time overnight against and additional 2 L of fresh 20 mM sodium phosphate buffer at pH 7.2 to remove trace amounts of tris buffer and sodium chloride still present in the sample via a second 2000-fold dilution. This reduced the concentrations of both tris and sodium chloride in solution to 2.5 nM and 37.5 nM respectively and therefore minimized their impact on CD measurements. |

### – CD analysis –

| Reviewer  Comment | and nothing is reported about the analysis of the collected data. |
| --- | --- |
| Author  Response | We analyzed the CD data in Figure 3, see the revised caption below. It is based on this analysis that we base our statements on coiled-coiling. |
| Excerpt From  Revised Manuscript | **Fig. 3. Mutations at position E174 affect θ_222_/θ_208_.**  *We plotted θ_222_/θ_208_, for temperatures from 20 to 92C. Ratios greater than unity typically indicate coiled-coils. θ_222_/θ_208_ did not exceed unity for E174A. θ_222_/θ_208_ lower than 0.9 indicates helices in isolation, [24] and indeed there is no clear coiled-coiling in experimental hGH structures (see PDB ID 1A22). Differences in ratio (comparing WT to E174A and E174Y) are greater at the lower end of the temperature range, presumably due to greater overall order. Linear regressions are shown for WT, E174A, and E174Y (L52F was left out for clarity) over the temperature range of 20 to 58C (in which θ_222_/θ_208_ varies linearly). Also shown are 95% confidence intervals for the regressions (generated by the seaborn regplot function, in light shading). E174A has clearly higher θ_222_/θ_208_ than E174Y and WT. E174Y has somewhat higher θ_222_/θ_208_ than WT. L52F is a control, as it is not in a helix and not near the zinc-binding triad; its θ_222_/θ_208_ is indistinguishable from that of WT, and is less than that of E174A. E174A / E174Y increase θ_222_/θ_208_ ratio, while L52F has no clear effect.* |

### – Table 1 and SPR –

| Reviewer  Comment | Where is table 1? The SPR results are not well described. |
| --- | --- |
| Author  Response | Table 1 was uploaded as a separate document. In the revised text, Table 1 is now in the main text document.  We agree it would have been hard to understand the SPR results and comparison to HomologyScanner without Table 1. The sensograms are provided as supplementary data. |
| Excerpt From  Revised Manuscript |  |

### – Discussion –

| Reviewer  Comment | 4) As a consequence of the point before also the discussion section needs to be revised, this paragraph is really poor, no correlations were reported between the methodologies used, just hypothesis and open questions are reported. Where is table 1? |
| --- | --- |
| Author  Response | The lack of correlation between calculation and experiment can be a bit deceptive. It is important to note that ONLY the substitutions with highest predicted affinity were expressed. This greatly restricts the range of ΔΔG_homologyScanner_ values, the lowest value is -2.54 kcal/mol, while most of the other substitutions range between -0.52 and -1.45 kcal/mol. In Flores et al. (2021), we report homologyScanner has a best-case RMS Error of 1.1kcal/mol – equal to the range of most of our data. For comparison, in Flores et al., ΔΔG_experimental_ data ranged from -6 to +7 kcal/mol, while ΔΔG_homologyScanner_ ranged from -2.5 to at least +5 kcal/mol (depending on specific dataset). This point is now made more clear in our revised Discussion.  Table 1 is now in the main text rather than a separate file. |
| Excerpt From  Revised Manuscript | Discussion  …  As noted in Table 1, there was no significant correlation between ΔΔG_homologyScanner_ and ΔΔG_experimental_. This could be expected because substitutions were selected for low ΔΔG_homologyScanner_ and so (aside from one -2.54 kcal/mol substitution) values range from -1.45 to -0.52 kcal/mol), over a small number of mutants. HomologyScanner has a reported [8] best-case Root Mean Square Error of 1.1 kcal/mol – about equal to the range of most of our data. SSIPe likewise showed no significant correlation. |

### – Conclusion –

| Reviewer  Comment | 9) The conclusions section is expected by the reader, but in this paper is not present, mainly because there is nothing to conclude. |
| --- | --- |
| Author  Response | A Conclusion section has been added. |
| Excerpt From  Revised Manuscript | Conclusion  In summary, we have investigated the mechanism of the unexpected increase in hGHR-binding affinity of hGH substitution E174A. We found that it actually decreases the on-rate but overcompensates by decreasing the off-rate to an even greater extent. The E174A mutation was found to induce coiled-coiling.  All substitutions that yielded affinities comparable to wild type also decreased both on-rate and off-rate, though in cases other than E174A, the net effect was to slightly decrease affinity. Four of the highest-affinity substitutions decreased mass compared to WT, suggesting that there was not enough space or flexibility in these helices to accommodate any increases in side-chain size. |

### – References –

| Reviewer  Comment | Minor issue:  1) Please renumbered the references, is difficult to consult them. |
| --- | --- |
| Author  Response | We have updated the reference list and confirmed that they are in the order first cited. |
| Excerpt From  Revised Manuscript |  |
